# Two Cases of Death from Chloroform

**Published:** 1869-05

**Authors:** 


Two Cases of Death from Chloroform have recently occurred in this city, in which every apparent care was taken to guard against such a result. We doubt not that there are yet to be found, despite many similar cases that are constantly occurring, many enthusiasts for this anæsthetic who are still ready to affirm that it has no direct agency in causing death. Such, however, can no more be convinced of their error than was the Indian who missed his way: "Indian no lost! only wigwam gone! !—*N. Y. Med. Record.*

